# Internal Hernia Through a Mesenteric Defect Following Esophagectomy and Reconstruction With a Stomach-Preserved Ileocolic Interposition

**DOI:** 10.7759/cureus.56244

**Published:** 2024-03-15

**Authors:** Daisuke Tomita, Kentoku Fujisawa, Yu Ohkura, Masaki Ueno, Harushi Udagawa

**Affiliations:** 1 Department of Gastroenterological Surgery, Toranomon Hospital, Tokyo, JPN

**Keywords:** postoperative complication, mesenteric internal hernia, ileocolon interposition, esophagectomy, case report

## Abstract

Esophagectomy is the standard treatment for esophageal cancer and often involves the stomach as a substitute organ for esophageal reconstruction. However, we actively perform stomach-preserved ileocolic interposition because of its advantages in gastrointestinal function and the prevention of reflux esophagitis. Despite its benefits, few facilities perform esophageal reconstruction with ileocolic interposition; hence, postoperative complications following this procedure have rarely been reported. We present the first case of internal hernia through a mesenteric defect following esophagectomy and reconstruction with a stomach-preserved ileocolic interposition. This type of internal hernia after esophageal cancer surgery is a rare complication following a common gastric pull-up reconstruction.

A 66-year-old Japanese female underwent esophagectomy and reconstruction with stomach-preserved ileocolic interposition for stage I esophageal cancer. One month after surgery, the patient experienced abdominal pain and vomiting. CT showed a dilated small bowel and a suspected postoperative adhesive bowel obstruction. Despite conservative management, the patient experienced recurrent episodes that required hospitalization. Although an exact preoperative diagnosis was not made, we decided on a surgical exploration six months after the first symptoms appeared. Laparotomy revealed an internal herniation through a mesenteric defect between the transverse mesocolon and the ileum mesentery following ileocolic interposition. We then repositioned the fitted small intestine and closed the mesenteric defects. The patient recovered uneventfully without a hernia recurrence.

Minimally invasive techniques for treating esophageal cancer are becoming more common. As survival rates improve, the number of internal hernia cases, such as those described in this report, will likely increase. Therefore, more cases are needed to determine whether closing mesenteric defects can effectively prevent herniation. However, immediate surgical treatment should be considered based on the symptoms, even when a preoperative diagnosis is difficult.

## Introduction

After esophagectomy for esophageal cancer, the stomach is usually the preferred organ choice for an esophageal substitute [1]. However, in patients with a history of gastrectomy or concurrent gastric disease, the ileocolon may be used instead [2]. At our hospital, we perform reconstruction using stomach-preserved ileocolic interposition because of its advantages in terms of gastrointestinal function, food reservoir function, and the prevention of reflux esophagitis [2]. Unfortunately, very few reports exist on postoperative complications following this procedure. Recently, we encountered a patient with an internal hernia through a mesenteric defect following esophagectomy and reconstruction with stomach-preserved ileocolic interposition. Therefore, we present this case, along with a literature review.

## Case presentation

**Figure 1:**
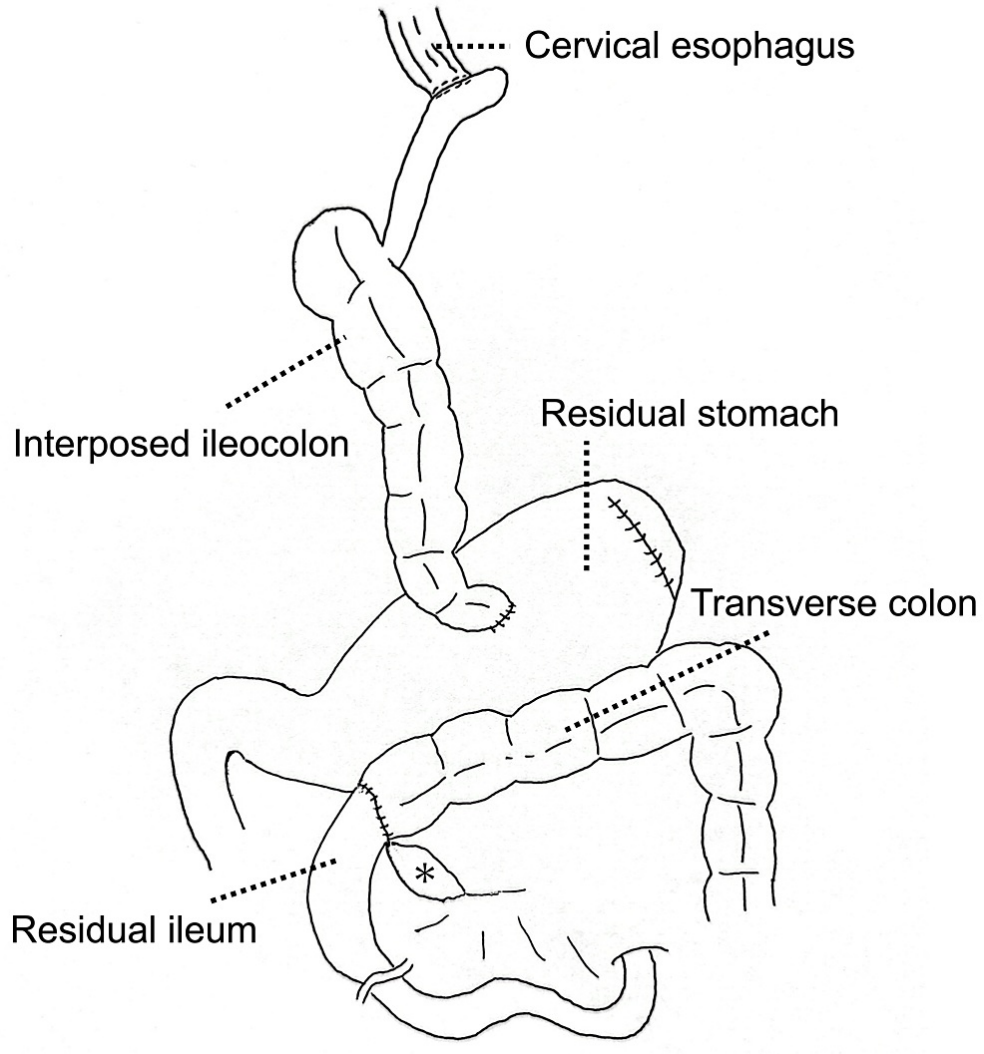
Design of the surgical reconstruction performed via the retrosternal route using ileocolon interposition *: mesenteric defect Image Credit: Author

The patient was a 66-year-old Japanese female who underwent esophagectomy via the right thoracoscopic approach and reconstruction with stomach-preserved ileocolic interposition via the retrosternal route. The patient was diagnosed with pT1bN0M0 Stage I cancer of the middle third of the esophagus. To perform ileocolic interposition, the region from the terminal ileum to the ascending colon was adequately mobilized from the retroperitoneum, which was performed laparoscopically. Transillumination and indocyanine green fluorography were used to examine the colic vessels. The ileocolon was then pulled to the neck via the retrosternal route. A cervical esophago-ileo anastomosis was performed in an end-to-side (hand-sawn) fashion. The distal end of the ileocolon was anastomosed to the anterior end of the residual stomach. The transverse colon was anastomosed to the residual ileum using the functional end-to-end anastomosis technique (Figure 1).

**Figure 2:**
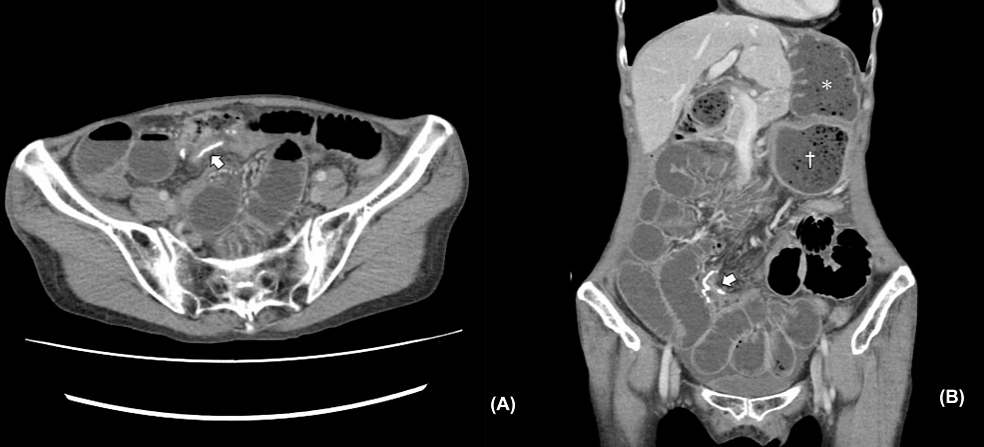
CT findings A small bowel obstruction was observed on a CT scan, but the cause is unknown. A: axial view, B: coronal view, arrow: anastomosis site of the residual ileum and the transverse colon, ＊: dilated interposed colon, †: dilated residual stomach, CT: computed tomography

**Figure 3:**
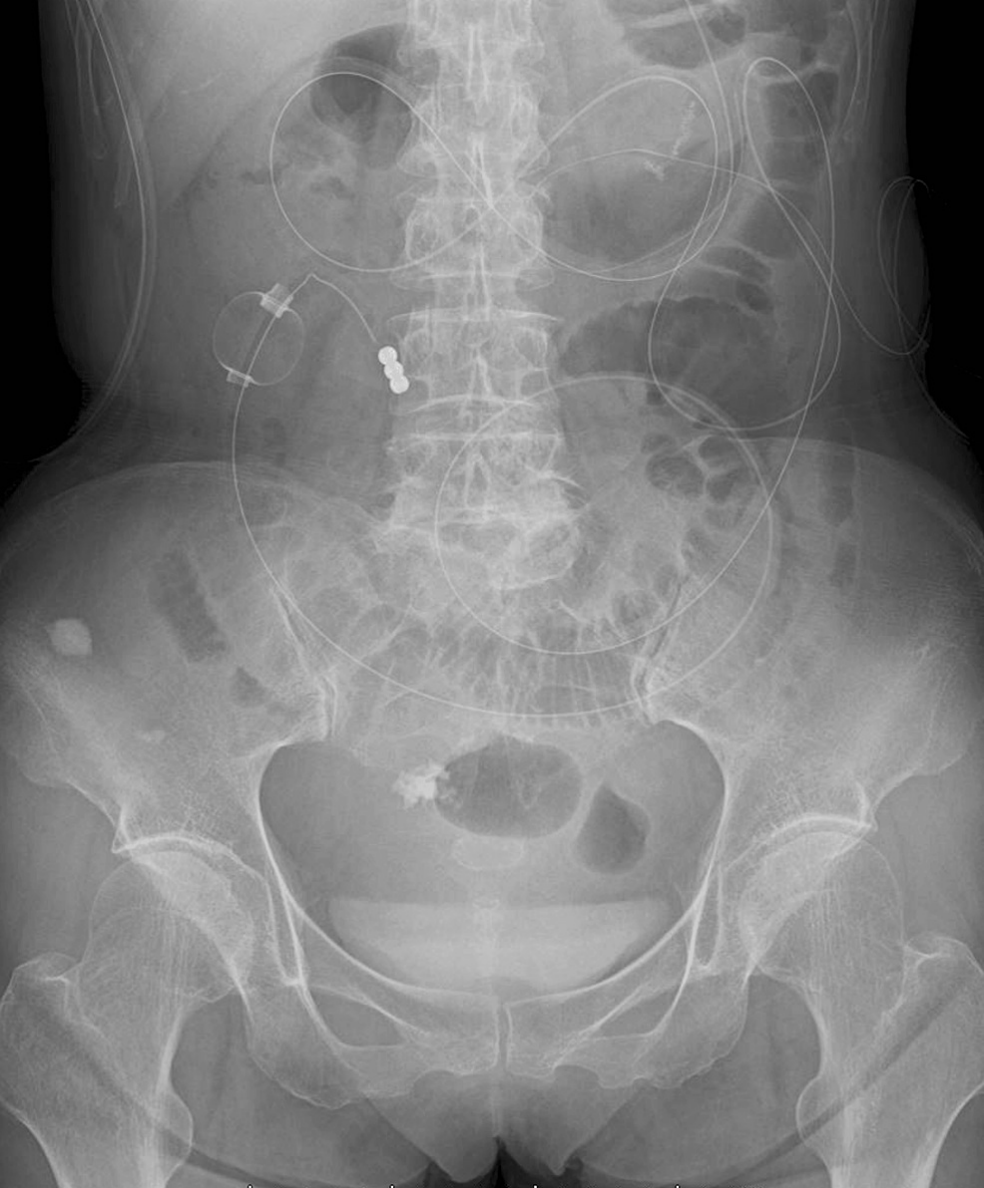
X-ray photograph after intubating a long tube A long tube was intubated into the dilated small bowel.

One month after the surgery, the patient experienced abdominal pain and vomiting. An abdominal X-ray showed a dilated small bowel with air-fluid levels. Abdominopelvic CT with intravenous contrast revealed a dilated interposed colon, residual stomach, and small bowel with air-fluid levels, but we were unable to identify an obvious origin of the obstruction (Figure 2A-2B). Since the contrast effect of the bowel was preserved and there were no findings suggestive of bowel ischemia or necrosis, we initially suspected a postoperative adhesive bowel obstruction. We decided on a conservative treatment plan, and a long tube was inserted into the dilated small bowel using endoscopy (Figure 3). Her symptoms improved within a few days, and she was discharged from the hospital. The patient experienced three instances of small bowel obstruction within six months, despite decompression with a long intestinal tube. The patient's condition was severe enough to require hospitalization three times in a short time span, indicating the need for surgery.

**Figure 4:**
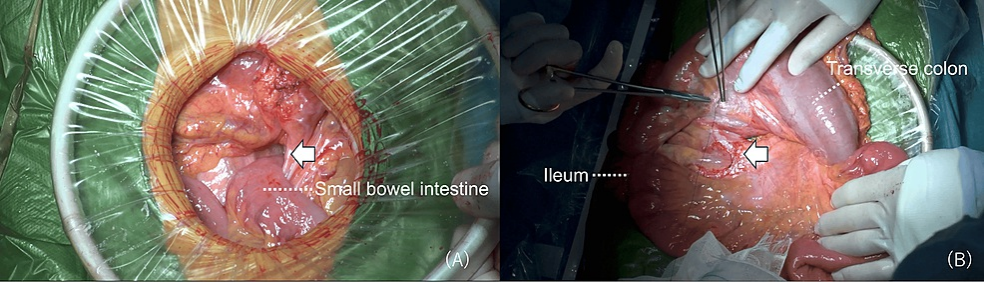
Intraoperative findings A: Small bowel intestine intrudes through the mesenteric defect (arrow) between the transverse mesocolon and the ileum mesentery. B: Closing the mesenteric defect (arrow) with suturing.

A laparotomy was performed. We found internal herniation of the small intestine through the mesenteric defect between the transverse mesocolon and ileum mesentery following ileocolic interposition (Figure 4A-4B). Therefore, we replaced the herniated small bowel and closed the mesenteric defect with suturing. All small bowels were confirmed to be free of ischemia and necrosis findings. The operative time was 74 minutes. The postoperative course of the patient was uneventful; she started eating on the third postoperative day and was discharged on the sixth postoperative day. Twenty-eight months have elapsed since surgery, and there has been no recurrence of either esophageal cancer or the internal hernia.

## Discussion

The stomach is typically used as a substitute when reconstructing the esophagus after esophagectomy for esophageal cancer because of its plasticity, ease of use, and rich submucosal vascular network [1]. However, if a patient has a history of gastrectomy, concurrent gastric disease, or cancer of the stomach, the colon is used instead of the stomach or jejunum. Our facility prefers using the ileocolon for several reasons [2]. Our previous report showed that ileocolonic interposition after esophagectomy with extended lymphadenectomy in patients with esophageal cancer is feasible and exhibits favorable outcomes [2]. The gastric retention capacity is preserved, and the cecum acts as a reservoir because the ileocecal valve protects against entering oesophageal reflux. The use of an ileocolonic graft was significantly associated with a reduced anastomotic leakage frequency. This is because the intestinal wall thicknesses of the esophagus and ileum are comparable and accessible to the anastomoses. Several studies have shown that this method is superior to gastric pull-up as an esophageal substitute in terms of the quality of life of patients with esophageal cancer [3-4].

Internal hernias are rare complications that occur after esophageal cancer surgery. The most common type of internal hernia after esophagectomy is hiatal hernia [5]. Studies have shown that the prevalence of post-esophagogastric surgery hiatal hernia is 3.18%, whereas that of hiatal hernia requiring surgical treatment is 2.01% [5]. Reports of other less common internal hernias in the retrosternal space have been published [6-8]. In recent years, minimally invasive techniques have become increasingly popular for esophageal cancer surgery. Although these techniques offer benefits such as decreased adhesion and greater organ mobility, they also increase the potential for herniation [9].

Even in high-volume centers, such as our institution, cases of internal hernia after esophageal cancer surgery are extremely rare. Our department performed 714 radical operations with primary reconstruction for thoracic esophageal cancer between January 2011 and December 2021. Of these surgeries, 186 underwent reconstruction with ileocolic interposition, 161 with stomach preservation, and 25 with previous or simultaneous gastrectomies. Of the patients who underwent ileocolic reconstruction, only nine (4.9%) developed postoperative bowel obstruction. Five patients were treated conservatively, whereas four required surgery. In all but one case, surgery was required because of the band formation associated with the adhesions.

To the best of our knowledge, this is the first reported case of internal herniation of the small bowel intestine through a mesenteric defect between the transverse mesocolon and small bowel mesentery after ileocolic interposition. This novelty may be because the ileocolon is rarely used as the first-choice reconstructive organ during esophagectomy. In this case, the small intestine prolapsed through the mesenteric defect caused by the anastomosis between the residual ileum and transverse colon, closely resembling an internal hernia after right hemicolectomy. Closure of mesenteric defects caused by anastomosis after colectomy is not routinely performed during laparoscopic surgeries. Cabot et al. reported that among 530 patients who underwent right hemicolectomy without closure of the mesenteric defect, only four (0.8%) experienced internal hernias with small intestinal insertion into the mesenteric defect, and that leaving the mesenteric defect open during laparoscopic right colon resection was not associated with a clinically significant incidence of internal hernia [10]. However, right hemicolectomy for malignancy is accompanied by lymph node dissection, and the mesenteric defect is considered sufficiently extensive to prevent the small bowel from fitting compared to ileocolic reconstruction in esophageal cancer, in which all the small bowel and colon mesentery are preserved. To avoid the occurrence of an internal hernia, closure of the mesenteric defect between the transverse mesocolon and the small bowel mesentery is an effective solution during esophagectomy with reconstruction using the ileocolon. However, given the risk of damage to the nutritional vessels of the reconstructed intestinal tract due to the closure of the mesentery and the very low incidence of internal hernia in the mesenteric defect, this may not be a realistic option.

Based on the patient’s symptoms, quickly determining whether surgery is necessary when bowel obstruction develops is crucial. Nausea and abdominal pain are often the primary complaints in the early stages of an internal hernia. Sometimes, this may progress to small bowel obstruction; therefore, prompt diagnosis is of utmost importance [11]. CT is useful for diagnosis as it can reveal characteristic findings of an internal hernia, such as the "whirl sign" of the mesenteric vessels due to axial torsion of the mesentery, mesenteric vessel abnormalities including crowding, stretching, and engorgement, and the lack of omental fat overlying the herniated bowel [12-14]. Surgical treatment should be performed promptly when spontaneous repair of an internal hernia is difficult. In recent years, surgical techniques have improved remarkably, and minimally invasive laparoscopic surgery is now being performed for small bowel obstruction [15]. If the previous surgery was performed laparoscopically and the intestinal tract is not dilated, laparoscopic surgery is a good indication for the treatment of internal hernias. Since more than 13% of internal hernia cases do not reach a definitive diagnosis on preoperative CT [16], laparoscopic surgery is also helpful as a diagnostic tool [17]. For this patient, although the previous abdominal operation was performed laparoscopically and the intestinal tract was not dilated at the time of reoperation, the surgery was performed by laparotomy because of concerns about damage to the reconstructed intestinal tract. The diagnosis could not be confirmed preoperatively in this case, but the decision to perform the operation was made because the small bowel obstruction was refractory to conservative therapy.

## Conclusions

Our patient exhibited a rare instance of an internal hernia through a mesenteric defect after ileocolic interposition. Minimally invasive approaches to treating esophageal cancer are likely to become more common in the future. As the long-term survival rate of postoperative patients with esophageal cancer improves, the number of reported cases of internal hernias, such as in this case, will likely also increase. Therefore, further accumulation of cases is necessary to determine whether closing the mesenteric defects can effectively prevent these hernias. However, if an internal hernia occurs, making a prompt diagnosis is essential. Even if the diagnosis is challenging, immediate surgical treatment should be considered based on the symptoms.
